# Real-World Implementation of Large Language Models for Writing Clinical Discharge Summaries Within a Secure Data Environment: Development and Expert Evaluation Study

**DOI:** 10.2196/88816

**Published:** 2026-07-03

**Authors:** Catalina Carenzo, Kathleen Goldsmith, Maite Arribas, Benjamin Atkins, Ina Ko, Ho Lun Chong, Asmita Raja, Aya M Riad, Rachael Lear, Yusuf S Abdullahi, Ben Glampson, Tim Orchard, Erik Mayer

**Affiliations:** 1 Imperial Clinical Analytics, Research and Evaluation (iCARE), NIHR Imperial Biomedical Research Centre Imperial College Healthcare NHS Trust London, England United Kingdom; 2 Department of Surgery & Cancer, Faculty of Medicine Imperial College London London, England United Kingdom

**Keywords:** artificial intelligence, AI, machine learning, natural language processing, large language models, generative pretrained transformer, GPT, clinical documentation, discharge summaries, electronic health records, clinical decision support, health information management, data security, expert validation study

## Abstract

**Background:**

A discharge summary should be a clinical report that documents a patient’s hospital stay, including test results, diagnoses, management, and follow-up. Currently, discharge summaries are written by clinicians who manually locate pertinent information across the electronic health record, of which approximately 80% is free text. This process is time-consuming and may be suitable for automation using large language models.

**Objective:**

This study developed a template-based prompting system that can produce clinically acceptable discharge summaries, specifically the “clinical summary” and “plan and requested actions” sections, from routinely collected electronic patient records.

**Methods:**

This study used electronic health record data from Imperial College Healthcare National Health Service Trust, a network of 5 hospitals in northwest London. It was conducted within the Imperial Secure Data Environment under Data Access and Ethics Committee approval. In total, 52 inpatient encounters were selected by the clinical team to ensure diversity in clinical specialty, reason for admission, complexity, length of stay, and sociodemographic characteristics; 83% (n=43) of the cases were allocated to the development dataset, and 17% (n=9) comprised the test dataset. The system synthesized clinical notes related to an inpatient hospital encounter and used structured template prompts with OpenAI’s generative pretrained transformer-4 to generate a discharge summary. The prompt was co-designed across 3 iterations. Resident physicians completed an evaluation form to assess the clinical acceptability of the generated summaries, including the primary outcome (global confidence rating) and secondary outcomes (accuracy, completeness, readability, formatting, sociodemographic bias, and potential clinical harm). Sensitivity analyses assessed the effect of length of stay and admission type (emergency department vs other and surgical vs other) on the primary outcome.

**Results:**

A total of 52 patients (n=32, 62% female) were included, with a mean age of 44.8 (SD 27.1) years and an average length of stay of 15.2 (SD 21.1) days. In the test dataset, 89% (8/9) of generative pretrained transformer-4–generated summaries received a positive global confidence rating (“yes” or “yes, with minor changes”). Secondary outcomes were positive for the “clinical summary” section (8/9, 89% complete and 7/9, 78% accurate) and the “plan and requested actions” section (7/9, 78% complete and 7/9, 78% accurate). Readability, formatting, sociodemographic bias, and potential clinical harm also showed positive results in the test dataset. Sensitivity analyses showed no statistically significant variation in the primary outcome across length of stay or admission type (length of stay: *P*=.29; surgical admission: *P*=.99; emergency department admission: *P*=.15).

**Conclusions:**

Our results demonstrate the feasibility of the pipeline, but rigorous statistical evaluation in a larger, adequately powered sample is needed.

## Introduction

The discharge summary is a critical clinical document that communicates a patient’s care in hospital, key clinical events, and plans for follow-up care. It typically includes patient demographics, hospital information, diagnoses, a clinical summary, medications, allergies, and pathology results. Currently, the responsibility for compiling the discharge summary often falls on a single clinician. This presents several challenges. First, inpatient care is often delivered by a multidisciplinary team of health care professionals [1], making it difficult for any single clinician to have complete oversight of all relevant clinical events and decisions. Second, the required information is dispersed across multiple documents within the electronic health record (EHR), including admission clerking notes, ward round entries, operation notes, and results (eg, pathology and radiology), making the process of locating and synthesizing key data time-consuming [2]. Third, EHR data are predominantly unstructured, with approximately 80% existing as free text [3], further complicating retrieval and summary generation. Finally, the volume of EHR data is continually increasing [4], intensifying the burden on clinicians tasked with producing accurate and comprehensive discharge summaries under time pressure.

Generative artificial intelligence (AI) refers to a class of AI that can create new content such as text [5] based on patterns learned from large datasets. A specific type of generative AI is the large language model (LLM) [6], which is trained to understand and generate humanlike text. In a clinical setting, LLMs have the potential to streamline documentation, automate routine tasks, and support decision-making within EHRs. A commonly used LLM is the generative pretrained transformer (GPT), which was first released by OpenAI in June 2018 [7] and has been continuously optimized into newer versions, including GPT-4, which we used in this study [8]. GPT has demonstrated the ability to generate humanlike text and can be adapted for specific tasks such as producing discharge summaries [9-18].

To leverage pretrained LLMs in clinical settings, prompt engineering has become increasingly central to research in developing clinical applications that minimize hallucination rate, preserve factual accuracy, and produce outputs for clinical use [19]. Prompt engineering is the deliberate design of inputs to generative models to align the output with the intended clinical application without the need for retraining [20,21]. A well-designed prompt provides structured and explicit cues to the model, facilitating accurate and efficient task completion. A range of different prompt patterns have been developed and applied in the context of text generation and summarization, and these techniques are increasingly being applied in clinical settings [22].

The use of LLMs to automate discharge summary generation has been examined using both synthetic or publicly available datasets [14-17] and real-world EHR data [9-13,18,23-25]. Among studies using real hospital data, several imposed constraints on the clinical cohort. Li et al [9] and Kim et al [12] restricted their populations to specific patient conditions [18] or to settings such as the emergency department (ED) [11] or narrowed inclusion by limiting the length of stay [10,13]. Ganzinger et al [23] relied on structured data that had to be manually extracted from semistructured and unstructured EHRs to construct the prompt context, underscoring the challenges of working with heterogenous clinical records.

This study aimed to develop a template-based prompting system that can produce clinically acceptable discharge summaries, specifically the “clinical summary” and “plan and requested action” sections, from unstructured, routinely collected electronic patient records. The system was iteratively co-designed by data scientists, engineers, and physicians. The system retrieved free-text clinical notes related to an inpatient hospital encounter and used structured template prompts to guide OpenAI’s GPT-4 in synthesizing the clinical documentation and generating a discharge summary that conformed to the UK Royal College of Physicians’ (London) 2021 guidelines [26] and National Health Service England’s information standard DAPB4042 [27]. The clinical acceptability of the generated summaries was evaluated using an assessment form completed by a clinical team of 4 resident physicians and included the primary outcome (physicians’ global confidence rating) and secondary outcomes (accuracy and completeness, clinical significance of incorrect or missing content, formatting, readability, and sociodemographic bias).

## Methods

### Setting and Data Source

This study was undertaken using EHR data from Imperial College Healthcare National Health Service Trust (ICHT), a large network of 5 hospitals providing acute and specialist care in northwest London serving over 1.3 million patients annually. The data were securely hosted in the Imperial Secure Data Environment (SDE), refreshed weekly since April 1, 2023. The Imperial SDE is approved to host identifiable and deidentified clinical and biomedical data. The ICHT instances in the SDE securely host routinely captured clinical information from the care delivered at ICHT, which includes clinical narratives that cover the full clinical record and patient pathways of all ICHT patients. The SDE was provisioned with an OpenAI model end point hosted on Microsoft Azure, with private network access made available to the data scientist from within the SDE. Azure Prompt flow was used to orchestrate prompt engineering, input data processing, and model interaction. The flow was configured to use OpenAI’s GPT-4 model (gpt-4-1106), which had a 128,000-token context window and an 80,000 token per minute limit.

All data used in this study were curated within the Imperial SDE and deidentified to remove any personal identifiable information, including any names and dates, before research access in line with the SDE processes. The ICHT data protection office and Caldicott guardian reviewed and approved the data and anonymization process. All infrastructure within the SDE was signed off by the ICHT security and data protection offices.

### Multidisciplinary Design Team Composition

The design team comprised postgraduate year 1 and 2 (equivalent to UK Foundation Programme year 1 and 2) resident physicians at ICHT with routine, hands-on experience writing discharge summaries working alongside data scientists and data engineers to iteratively co-design the system.

### Service Evaluation

A service evaluation was conducted (ICHT approval 906) where researchers and technical members of the team observed resident physicians completing discharge summaries. This provided insights into the types of documents accessed within the EHR; the order in which they were reviewed; the specific information extracted; and factors considered relevant when assessing a discharge summary, including accuracy and completeness. Documents that were identified as relevant for writing a discharge summary included admission clerking, ward round notes, specialty reviews, operation notes, and results.

### Sample Selection

A total of 52 inpatient encounters, each corresponding to a unique patient, in which the resident physicians had provided direct care at ICHT between April 1, 2023, and December 5, 2023, were manually selected by the resident physicians (a unique patient encounter will be referred to as a “case” hereafter). The cases were chosen to ensure diversity in clinical specialty, reason for admission, complexity, length of stay, and sociodemographic characteristics. Additionally, cases were selected for which there was a discharge summary documented in the EHR.

**Figure 1:**
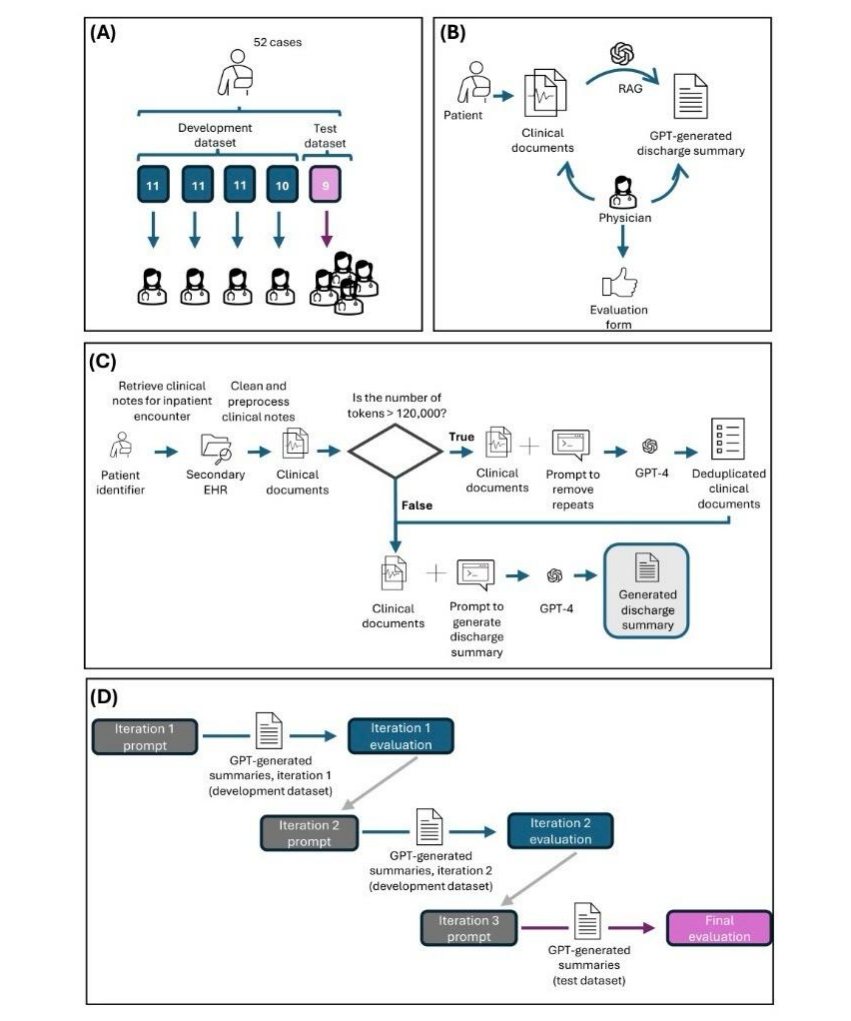
Infographic summary of the methods. (A) 52 cases divided into 5 groups matched on mean length of stay. One group served as the left-out test set (pink); the remaining 4 formed the development set (blue), each assigned to one resident physician for evaluation in iterations 1 and 2. All 4 physicians reviewed test cases. (B) Flowchart showing how clinical documents and GPT–generated discharge summaries were used by physicians to complete the evaluation form for each case. (C) Context preprocessing pipeline (see Methods section “Prompt-Templated Pipeline Development”). (D) Prompt engineering pipeline: prompts from iterations 1 and 2 generated summaries on the development set; the iteration 3 prompt was applied to the test dataset. EHR: electronic health record; RAG: retrieval-augmented generation.

The 52 cases were first divided into 5 subsets, each containing a similar number of encounters and a comparable mean length of stay, which was used as a proxy for clinical case complexity. Four subsets (n=43, 83% of the encounters) were designated as the development dataset, whereas the fifth subset (n=9, 17% of the encounters) was held out as an independent test dataset. This test dataset was isolated prior to model development and was not accessed during prompt design, system development, or iterative testing. An infographic describing this process is shown in Figure 1A.

After iteration 1, each physician selected the single best-performing case from their assigned development subset to serve as a few-shot example. This resulted in 4 examples that were incorporated into the few-shot learning prompt used in iterations 2 and above (see Multimedia Appendix 1 for details). Consequently, for iteration 2 onward, the development dataset size was reduced from 43 to 39 cases.

### Prompt-Templated Pipeline Development (3 Iterations)

#### Data Preparation

An overview of the pipeline, evaluation process, and iterative development is provided in Figures 1B-1D. For each selected case, all clinical documents associated with the encounter ID were selected and preprocessed. Data preprocessing removed HTML tags, special characters, and irrelevant sequences of words and sentences (eg, “Report starts/ends here”). The Python libraries Pandas, Beautiful Soup, and RegEx were used to facilitate this process.

After iteration 1, some clinical notes were excluded from the selection. The service evaluation concluded that certain clinical notes were identified as less directly informative for drafting a discharge summary and so were excluded from the knowledge base for all cases, with agreement from the 4 resident physicians. These included some notes labeled as nursing notes, board rounds, and other entries with specific tags or phrases. Additionally, the resident physicians and data scientists put together a list of all possible discharge summary–type documents, and this was used to remove all discharge summary–type documents from the corpus of clinical notes and hold them separately for use in the evaluation of the pipeline. For each patient encounter, cleaned clinical notes were put into the order in which they were created and then combined into one piece of text for use in the case-specific prompt.

#### Data Chunking

In some cases, the total number of tokens of the clinical notes combined with the prompt and output token limit exceeded the overall token limit for GPT-4, which is 128,000 tokens. For these documents, an additional Prompt flow program was used that divided the text into 5 smaller chunks, which overlapped by 1000 tokens. These chunks were then sequentially passed via a prompt to the GPT-4 model, where the prompt instructed it to remove duplicated pieces of information without changing the words or order of the original text. The output of these 5 separate prompts was then combined into a single document, and this document replaced the original document of all clinical notes for that case (Figure 1C).

#### Indexing

For each case, the resulting combined document was stored as a .txt file in an instance of Azure AI Search. To use the keyword search functionality at inference, each document had the patient identifier number entered at the start of the document. At inference, this patient identifier was used to search the index for the clinical notes relevant to that patient encounter.

#### Prompt Engineering and Model Parameters

The resident physicians and the data scientist co-designed the prompt. Using the development dataset, the system message and different prompting patterns were explored to understand how they affected the generated text, as outlined in Multimedia Appendix 1.

#### Content Moderation

The default content moderation system applied to GPT-4 end points was blocking prompts containing language classified as violent, which led to refusals during inference. To resolve this, a custom content moderation configuration was deployed on the ICHT model end points.

#### Evaluation During Development

Each of the 4 subsets of cases from the development set was randomly assigned to a resident physician for evaluation during iterations 1 and 2. For each case, the physicians were provided with the clinical notes that had been used as inputs to the prompts and the GPT-generated summary.

For each case, the physician completed an evaluation form in a Microsoft Excel sheet. The evaluation form covered a set of questions, including the primary and secondary outcomes. The primary outcome was defined as the physician’s global confidence rating (“Based on the quality of the discharge summary, would you be willing to sign your name to it?” Response options: “yes,” “no,” and “yes, with minor changes”). The secondary outcomes included accuracy and completeness, clinical significance of incorrect or missing content, formatting, readability, and sociodemographic bias (see Table S1 in Multimedia Appendix 1 for further details).

### Evaluation (Test Dataset)

The final prompt developed from iteration 3 was used to generate discharge summaries for the 9 hold-out cases in the test dataset. These cases were randomly assigned to the resident physicians, with each physician reviewing at least 2 cases and completing an evaluation form for them.

### Statistical Analysis

Due to the limited sample size (N=52) and power, statistical comparisons across iterations were not computed. Instead, the results from the evaluation form in iterations 1 and 2 (development dataset) and the final evaluation (test dataset) are visualized as stacked bar charts. The frequency of binary and categorical responses for the primary and secondary outcomes is reported.

### Sensitivity Analysis

To examine whether length of stay (measured in days as a continuous variable), emergency department admission (yes or no), or surgical admission (yes or no) were associated with the primary outcome of global confidence rating (categorical: “yes,” “no,” and “yes, with minor changes”), we ran 3 separate Fisher-Freeman-Halton exact tests in R version 4.4.1. Statistical significance was assessed at the .05 level. For the sensitivity analysis, data from the development dataset (iteration 2) and test dataset (final evaluation) were pooled to maximize statistical power.

### Ethical Considerations

This study was undertaken within the Imperial SDE and received approval from the Data Access and Ethics Committee. The Imperial Clinical Analytics Research and Evaluation (iCARE) research database was given favorable ethics approval by the South West – Central Bristol Research Ethics Committee (reference 21/SW/0120; Integrated Research Application System project ID 282093). All data used in this paper were effectively anonymized before analysis. The systems and processes were reviewed and approved by the ICHT security and data protection offices.

## Results

### Patient Sample

A total of 52 patients were included in this study (Table 1), with a mean age of 44.8 (SD 27.1) years at admission. In total, 62% (n=32) of the sample were female, with the most common ethnicity being other (n=21, 40%), followed by White—British or Irish (n=12, 23%), White—other (n=8, 15%), Black (n=6, 12%), Asian (n<5, <10%), and mixed (n<5, <10%). The average length of hospital stay was 15.2 (SD 21.1) days. Most patients (n=38, 73%) were admitted via the ED, with a smaller proportion (n=15, 29%) being admitted via the surgical department. The distribution of patients' *International Classification of Diseases* diagnoses and the specialties they received care from during their hospital encounter are shown in Tables S2 and S3 in Multimedia Appendix 1.

**Table 1:** Summary of the sociodemographic and clinical variables for the total sample (N=52).

Characteristic			Values
**Age at admission (years), mean (SD)**			44.8 (27.1)
	Missing	0 (0)	
**Sex assigned at birth, n (%)**			
	Female	32 (62)	
	Male	20 (38)	
	Intersex	0 (0)	
	Missing	0 (0)	
**Ethnicity, n (%)**			
	Asian—any other Asian background	<5 (<10)	
	Black or Black British—African or Caribbean	6 (12)	
	Mixed—any other mixed background	<5 (<10)	
	White—British or Irish	12 (23)	
	White—any other White background	8 (15)	
	Other	21 (40)	
	Missing	0 (0)	
**Length of stay (days), mean (SD)**			15.2 (21.1)
**ED^a^ admission, n (%)**			
	Yes	38 (73)	
	No	14 (27)	
	Missing	0 (0)	
**Surgery admission, n (%)**			
	Yes	15 (29)	
	No	37 (71)	
	Missing	0 (0)	

^a^ED: emergency department.

### Primary Outcome

**Figure 2:**
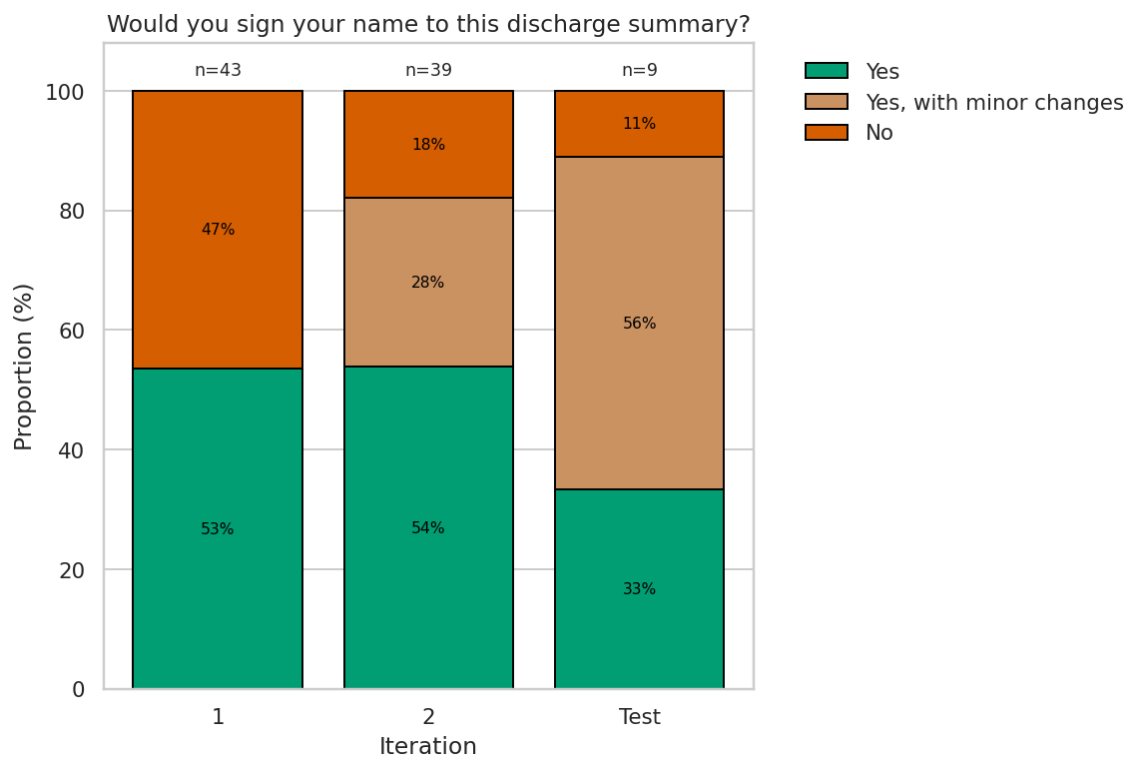
Stacked bar chart showing the primary outcome (global confidence rating) across iterations (development dataset) and the final evaluation (test dataset). Green indicates “yes,” light orange indicates “yes, with minor changes,” and dark orange indicates “no.” The possible answers to the global confidence rating question in iteration 1 were “yes” and “no.” The option “yes, with minor changes” was introduced in iteration 2 and testing due to an understanding that a discharge summary tool in practice would allow for minor edits.

The primary outcome, global confidence rating, across iterations (development dataset) and the final evaluation (test dataset) is shown in Figure 2, with the confidence interval (CI) for the proportions shown in Table 2. A greater proportion of GPT-generated discharge summaries received a “yes” or “yes, with minor changes” sign-off from resident physician reviewers than received a “no.” The proportion of negative responses (“no”) decreased from iteration 1 to iteration 2 (20/43, 47% to 7/39, 18%), suggesting that the prompt modifications between iterations 1 and 2 may have improved the quality of the generated summaries. However, it is important to note that the option “yes, with minor changes” was introduced only in iteration 2 alongside the original binary responses (“yes” or “no”). Moreover, evaluation on a held-out test dataset (with no data leakage) revealed a similar pattern, with a minority of GPT-generated discharge summaries (1/9, 11%) declined sign-off by the reviewer.

**Table 2:** Proportion of observations for the primary outcome (global confidence rating) across iterations 1 and 2 (development dataset) and the final evaluation (test dataset).

Would you sign your name to this discharge summary?	Iteration 1 (n=43 observations), n (%; 95% CI)	Iteration 2 (n=39 observations), n (%; 95% CI)	Test (n=9 observations), n (%; 95% CI)
No	20 (47; 33-61)	7 (18; 9-33)	1 (11; 2-43)
Yes, with minor changes	N/A^a^	11 (28; 17-44)	5 (56; 27-81)
Yes	23 (53; 39-67)	21 (54; 39-68)	3 (33; 12-65)

^a^N/A: not applicable.

### Sensitivity Analyses

No statistically significant association was observed between global confidence rating (“Yes”, “Yes with minor changes”, “No”) and any of these variables (length of stay: *P*=.29; surgical admission: *P*=.99; emergency department admission: *P*=.15). These findings suggest no statistically significant variation in global confidence ratings across these patient variables (Figure S1 in Multimedia Appendix 1).

### Secondary Outcomes

**Figure 3:**
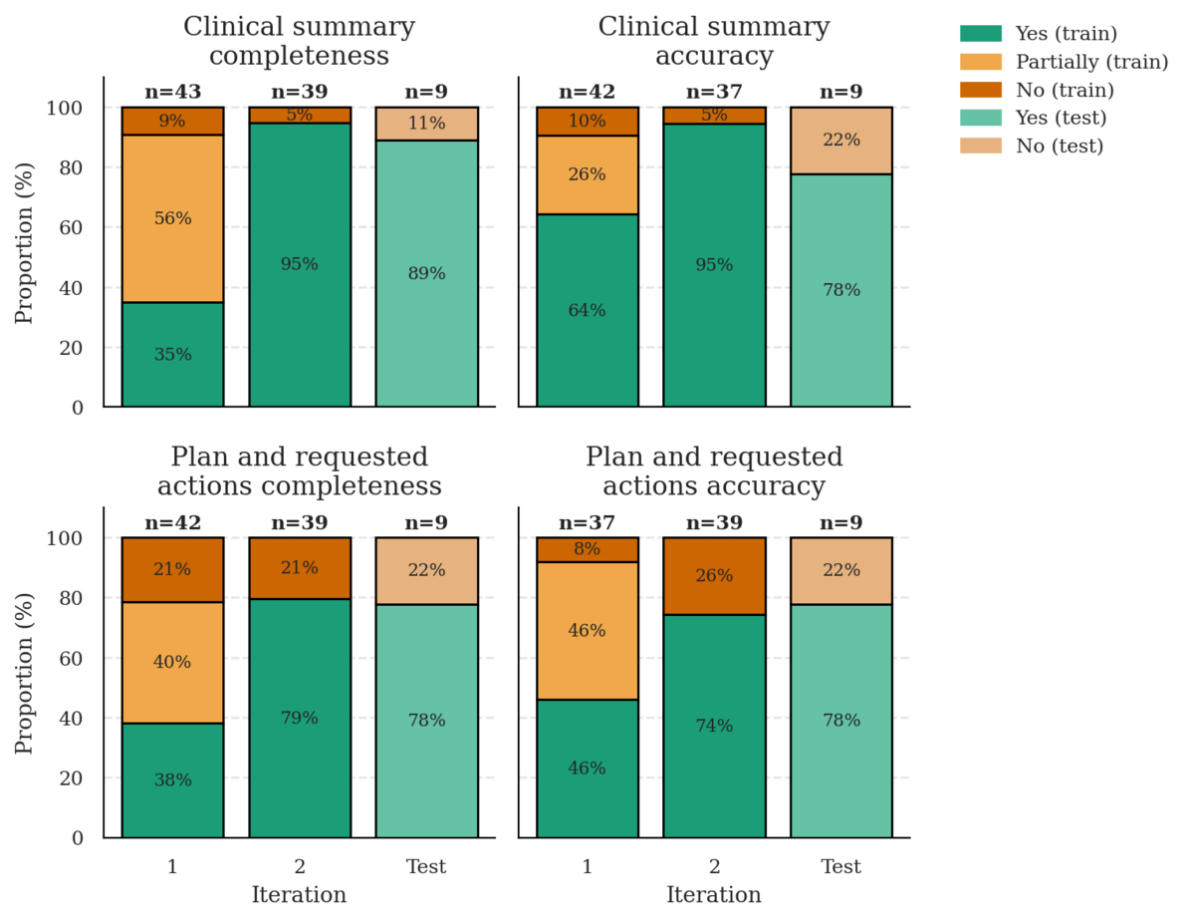
Stacked bar chart showing the accuracy and completeness of the (1) “clinical summary” and (2) “plan and requested actions” sections across iterations (development dataset) and the final evaluation (test dataset). Green indicates “yes,” light orange indicates “partially”—an option removed from iteration 2—and dark orange indicates “no.” The lighter colors correspond to the test dataset.

Accuracy and completeness of the (1) “clinical summary” and (2) “plan and requested actions” sections of the GPT-generated discharge summaries are shown in Figure 3. For the “clinical summary” section, most responses across iterations 1 and 2 were positive (39/43, 91% and 37/39, 95% complete; 38/42, 90% and 35/37, 95% accurate). For the “plan and requested actions” section, most responses were also positive across development iterations (33/42, 79% and 31/39, 79% complete; 34/37, 92% and 29/39, 74% accurate). In the test dataset, ratings were consistent with those of iteration 2, with positive results for the “clinical summary” section (8/9, 89% complete and 7/9, 78% accurate) and the “plan and requested actions” section (7/9, 78% complete and 7/9, 78% accurate).

**Figure 4:**
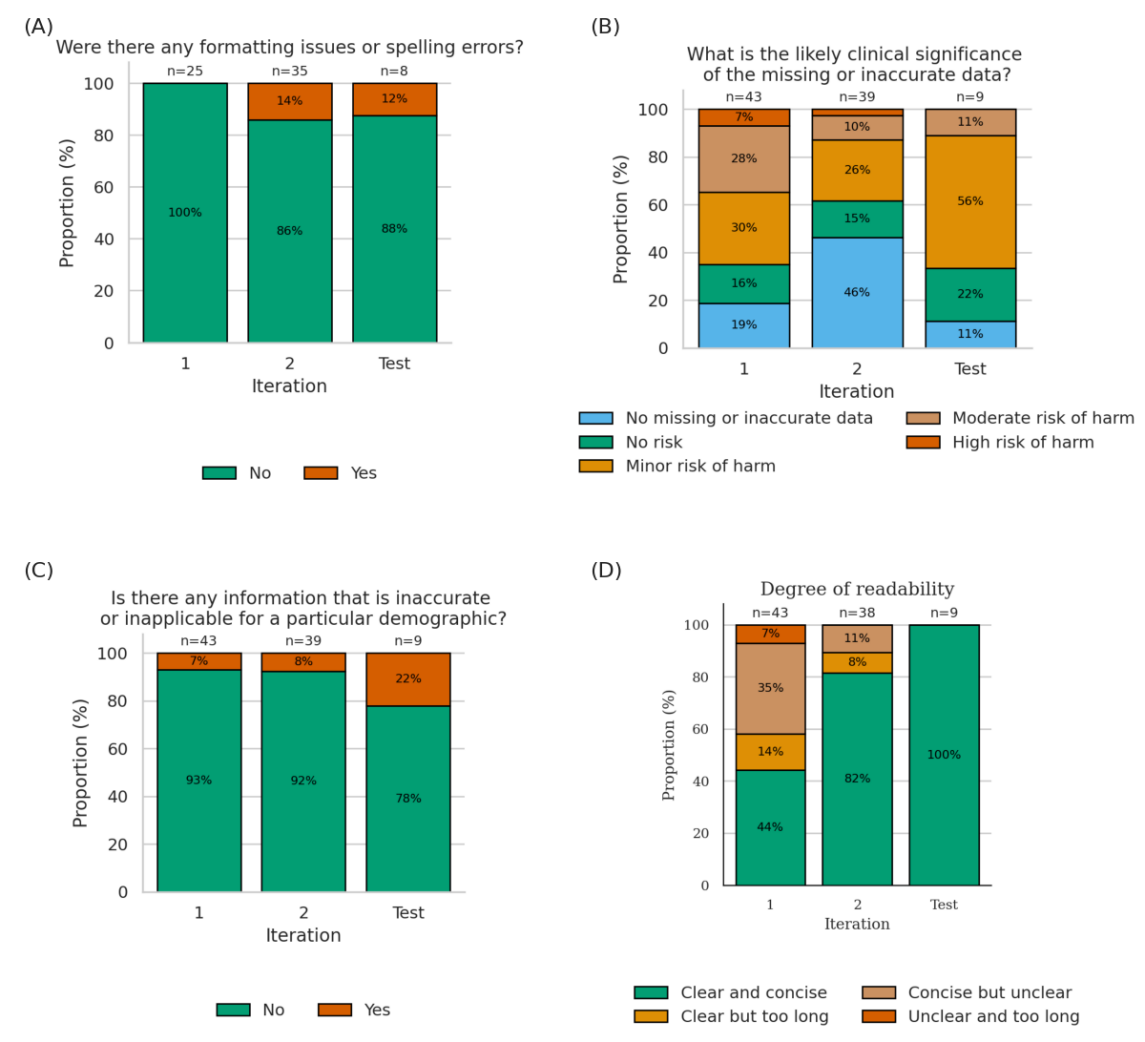
Stacked bar charts showing formatting issues (A), the clinical significance of inaccurate or missing information (B), sociodemographic bias (C), and readability (D) across 2 development iterations and the final evaluation (test dataset).

Other secondary outcomes included the clinical significance of inaccurate or missing information, sociodemographic bias, formatting issues, and readability (Figure 4).

For the clinical significance of inaccurate or missing information, there was a slight improvement from iteration 1 to 2, with a reduced proportion of ratings of high or moderate risk. In the test dataset, no summaries were rated as having a high risk, and most responses were rated as having no or minor risk (8/9, 89%). For sociodemographic bias, most responses across iterations 1 and 2 were positive (40/43, 93% and 36/39, 92% reported no bias); in the test dataset, this dropped to 78% (7/9). For formatting issues, most responses across both development iterations were positive (25/25, 100% and 30/35, 86% no formatting issues), with the same reflected in the test dataset (7/8, 87%). For readability, there was a considerable improvement from iteration 1 to 2 (19/43, 44% to 31/38,79%), with all responses being “clear and concise” in the test dataset.

## Discussion

We present the development of a prompt-templated pipeline for generating discharge summaries using OpenAI’s GPT-4 model [8] from routinely collected patient EHRs. From our prompt engineering iterative process, our final prompt used examples of high-quality input-output pairs for few-shot learning. The utility and clinical safety of the generated discharge summaries was evaluated by resident physicians. Our results demonstrate the potential effectiveness of this system, with evaluations by resident physicians showing a majority (8/9, 89%) of positive ratings on the primary outcome (global confidence rating), as well as on secondary outcomes (readability, formatting, sociodemographic bias, and potential clinical harm). However, rigorous statistical evaluation in a larger, adequately powered sample is needed [23]. As discussed below, this study presents a series of innovations relative to the work previously done in this field.

First, by harnessing the Imperial SDE [28], the data used to generate discharge summaries in this study came from unstructured, real-world EHRs. This constitutes a significant advancement in the field, which has largely been limited to synthetic or publicly available datasets or structured data extracted from the EHR [14-17]. Moreover, the prompt was designed and refined by a multidisciplinary team of resident physicians, data engineers, and data scientists through an iterative approach informed by the Royal College of Physicians discharge summary guidelines [27]. The physicians’ domain expertise and active involvement ensured that the system was not only performant but also aligned with the practical realities of clinical workflows.

Second, although the final evaluation was limited to a small sample (n=9), we found potentially promising results for the clinical acceptability of the generated outputs, aligning with previous research [9,13]. For example, 100% (9/9) of our discharge summaries were rated as clear and concise relative to lower ratings in previous studies (eg, Cai et al [29] observed that only 39% of the summaries obtained a full score on readability). The high readability score could be attributed to aspects of our pipeline, such as the low value of the top_p parameter, which reduces the use of excessive text decorations and ensures that the summary remains concise and focused on the relevant medical information. Moreover, the prompt specified that medical acronyms in the text should be translated to their expanded term following the Unified Medical Language System, which could reduce the ambiguity of medical acronyms [30].

Third, in addition to the prompt design, we showed that the selection of input data for the prompt template is important and can affect the quality of the discharge summary output. This was previously shown by Hartman et al [31], where progress notes and consults were the primary source of content selected for generating a summary of a patient’s hospital stay, with other documents being removed. We carried out a service evaluation with clinical experts to identify the key inputs needed for effective discharge summary generation. These included admission clerking, ward round notes, specialty reviews, operation notes, and diagnostic results (eg, pathology and radiology). Due to a proportion of the clinical notes in the EHR being of unknown type, such as blank notes (a text file without any preformatting or title), clinical notes were filtered by excluding those deemed irrelevant for drafting a discharge summary, such as nursing notes and board round notes. By using this cleaner set of input documents in iteration 2, improvements were observed in the discharge summary evaluation for the primary outcome compared to iteration 1 (28% more responses were positive for the global confidence rating), as well as some secondary outcomes (eg, the “clinical summary” section improved by 4% on completeness and 5% on accuracy, and readability improved by 37%). However, it is worth noting that these improvements may also be attributed to any changes to the prompt across iterations.

This study presents several avenues for future research. First, the evaluation of LLM-generated outputs can be further expanded. For example, for a proportion of cases in both iteration 2 and the test dataset (9/37, 24%; 3/9, 33%; Multimedia Appendix 1), resident physicians stated that they needed to consult with the team that treated the patient due to insufficient data in the clinical notes. This corresponds with the work by Ando et al [24], who found that 39% of the discharge summary information was sourced externally to the EHR, with up to 11% derived from the clinician’s memory. To further understand the need for a human in the loop, additional metrics such as gap distance could be introduced in the evaluation form, as suggested by Cai et al [29]. In addition to qualitative evaluation, quantitative methods such as the Recall-Oriented Understudy for Gisting Evaluation score [32], bidirectional encoder representations from transformers score [33], and Flesch-Kincaid readability ease [34] provide an approach for standardized evaluation of LLM-generated outputs.

To move toward the safe implementation of our pipeline into a real-world clinical setting, several challenges, such as model bias and fairness [35], would need to be addressed, as outlined in previous studies [36]. Moreover, a better understanding of the clinical scenarios where the tool could be implemented successfully is required. Case complexity often results in a higher number of documents, which poses a challenge due to the limited effective context window of LLMs [37]. To address this, future research should explore methods to improve the selective preprocessing of input data. Research could also stratify cases by factors indicative of case complexity, such as treatment specialty, care transitions, or length of stay. We explored some of these in our sensitivity analysis, but a larger sample would be necessary to inform data-driven clinical guidelines.

Safe implementation in practice would also require careful attention to data privacy, transparency, and model governance. In this study, data were deidentified, and the model was deployed through Azure OpenAI within a managed virtual network inside the Imperial SDE, providing network isolation and controlled access. Prompts and outputs were processed only for inference and were not used to train the underlying foundation models. If implemented in routine clinical practice, similar technical safeguards would need to be complemented by transparent documentation and clear governance around proprietary model updates. As proprietary LLMs such as the GPT series are typically subject to vendor-driven version changes, mechanisms for version control, auditability, and ongoing performance monitoring would be necessary to ensure that model updates or changes in clinical data environments do not adversely affect reliability, reproducibility, or patient safety.

There are several limitations worth considering. First, missing data present a challenge for LLMs [24]. Task completion is rarely documented by physicians in EHRs, leaving the model to infer which actions require follow-up in discharge plans, often resulting in inaccuracies. Similarly, some remote specialist or senior input, such as phone consultations, is frequently underdocumented or even omitted. Moreover, due to anonymization preprocessing in our study, key details such as time stamps and locations were removed. This hindered the model’s ability to sequence clinical events chronologically. However, this issue would be mitigated in a real-world clinical setting, where the data are not anonymized. Second, different specialties often use inconsistent formats, and plans can sometimes conflict, such as between renal and cardiology teams. In some cases, the model struggled to generate unified discharge plans. Moreover, medical abbreviations can be ambiguous, and their meaning may vary based on context and specialty (eg, “PT” could refer to physiotherapy, prothrombin time, or patient). Directly integrating the model with a terminology or abbreviation database such as the Unified Medical Language System could help improve its interpretative accuracy. Third, given that general practitioners and patients are the key readers of discharge summaries, their evaluation of the GPT-generated summaries would also be valuable. Fourth, the small sample of discharge summaries limits our ability to formally assess the clinical acceptability of the generated summaries or potential sources of bias. In addition, cases were manually selected by clinicians who had directly cared for the patients; therefore, the sample was not representative of the broader patient population and may be subject to selection bias. For the purposes of this study, which primarily aimed to codevelop an effective prompt, resident physicians were asked to select cases with a range of lengths of stay to capture varying levels of clinical complexity. In addition, interrater reliability was not assessed because each case was evaluated by a single physician. Future work should evaluate a larger set of generated discharge summaries across a representative sample and include assessment of interrater reliability and whether specific patient, clinical, or contextual factors systematically influence the quality. However, this would be very resource intensive due to the time-consuming aspect of discharge summary evaluation. For example, in our study, clinician evaluation took approximately 10 minutes per case. As proposed by Gero et al [38], LLM self-verification tools could be used to show the source of the input data used in the discharge summary generation and, thus, shorten the time required for physicians to review the input notes.

## Data Availability

The datasets generated or analyzed during this study are not publicly available due to the data being held in the Imperial Secure Data Environment (SDE) and therefore being subject to information governance and data protection restrictions. It may be made available from the corresponding author on reasonable request.

## Appendix

Multimedia Appendix 1 Prompt engineering methods and model parameters, the evaluation assessment form, sample International Classification of Diseases, Tenth Revision diagnoses and hospital specialties for the cohort, proportions and 95% CIs for secondary outcomes across iterations, and a figure of primary outcomes stratified by length of stay, surgical admission, and accident and emergency admission.

## Abbreviations

AI: artificial intelligence
CI: Confidence Interval
ED: emergency department
EHR: electronic health record
GPT: generative pretrained transformer
ICHT: Imperial College Healthcare National Health Service Trust
LLM: large language model
SDE: Secure Data Environment
