# Supplementary material for: Real-World Implementation of Large Language Models for Writing Clinical Discharge Summaries Within a Secure Data Environment: Development and Expert Evaluation Study
Source: JMIR AI. 2026 Jul 3;5:e88816. doi: 10.2196/88816 (PMC13379688; doi:10.2196/88816)
Supplement: Multimedia Appendix 1 [file ai_v5i1e88816_app1.docx]

**SUPPLEMENTARY MATERIAL**

**Supplementary Methods 1** Prompt Engineering and Model Parameters

The full prompt templates used in the iterations and for the final test set are not included in the manuscript due to licensing and copyright associated with the prompt framework. However, the structure of the prompt, model parameters, and retrieval configuration are described below. Researchers interested in reproducing the experiments may apply to access the complete prompt templates from the Imperial Secure Data Environment.

Please contact [imperial.DCS@nhs.net](mailto:imperial.DCS@nhs.net) to request access to the complete prompt template.

**Iteration 1**

**Prompt text** was designed to specify the persona of the prompt, provide clear instructions defining the scope of each section of the clinical summary, and establish the structure and format of the output to enhance the quality of the results. The system message was formatted to align with the associated roles (system, user, and assistant) and utilised indexed data as supporting content for generating the clinical summary. Additionally, the instructions were reiterated at the end of the system message to ensure consistency and adherence to the guidelines.

**Prompt technique:** Zero-shot learning

**Model parameters:** Temperature 0, top_p 0.95, frequence penalty 1, max tokens in output 4096.

**Context included in the prompt:** All unstructured clinical notes associated with the inpatient admission were retrieved using the encounter identifier linked to each case.

**Iteration 2**

**Prompt text** was broken down into smaller subtasks using a chain-of-thought approach. designed to specify the persona of the prompt, provide clear instructions defining the scope of each section of the clinical summary, and establish the structure and format of the output to enhance the quality of the results. More examples were provided to help the model interpret the phrasing commonly used by resident doctors, along with additional context to clarify what content should appear in each section. Finally, explicit emphasis was placed on avoiding the interpretation of unclear results and the length of the Safety Netty Advice section. Changes were implemented with repeated iterations on the development set with review from the resident doctors and the data scientist.

**Prompt technique:** Few-shot learning was implemented using the 4 best examples from iteration 1, each identified by a resident doctor.

**Model Parameters:** Temperature 0, top_p 0.5 frequence penalty 1, max tokens in output 4096.

**Context included in the prompt:** All unstructured clinical notes associated with the inpatient admission were retrieved using the encounter identifier linked to each case. Notes labelled as nursing notes, board rounds, or other entries containing specific tags or phrases that were collectively identified as less directly informative for discharge summary generation were excluded.

**Test**

**Prompt text:** specifications were added to reference the use of the Unified Medical Language System. Certain phrasing was refined, such as changing 'This section shouldn’t contain...' to 'This section must only contain...' to ensure clarity and precision. Additionally, examples of inpatient plans were included for each selected example used in few-shot learning, further reducing ambiguity and improving the model's interpretability.

**Prompt technique:** Few-shot learning as in iteration 2.

**Model Parameters:** Temperature 0, top_p 0.95, frequence penalty 1, max tokens in output 4096.

**Context included in the prompt:** As in iteration 2, all unstructured clinical notes associated with the inpatient admission were retrieved using the encounter identifier linked to each case. Notes labelled as nursing notes, board rounds, or other entries containing specific tags or phrases that were collectively identified as less directly informative for discharge summary generation were excluded.

**Supplementary Table 1^[[1]](#endnote-2)^** Evaluation Assessment Form^1^

| What is the case ID For the discharge summary you are reviewing? |  |
| --- | --- |
| **General considerations** | |
| Is the clinical summary complete?  Brief summary of the encounter, which may include: - Admission details & admission method. - Interpretation of findings and results. - Diagnoses (diagnosis confirmation, active diagnosis being treated) - Procedures (procedure name, anatomical site, laterality, complication, anaesthetic issues) - Safety alerts (including risks to self & others, mental capacity assessment, advanced decision making, lasting power of attorney, safeguarding issues).  (Incomplete: The clinical summary omits any information that it should not omit) | [Yes/No/Partially] |
| Is the plan and requested actions section complete? May include: - Actions for health professionals - Investigations requested - Procedures requested - Follow up appointments - Information given to patients: self-care and lifestyle advice - Safety netting advice & when to return - Resus status if changed during admission (Incomplete: The plan and requested actions omit any information that it should not omit) | [Yes/No/Partially] |
| Is the omission(s) related to a problem with: | [The source data/The system message/Both/Other] |
| If selected other in the previous question, please complete: | [free-text] |
| Provide details of the problem and suggest a solution e.g. "Amend system prompt as follows 'Please include safeguarding concerns, where relevant"". | [free-text] |
| Any formatting issues, spelling errors, etc? | [Yes/No] |
| Is the clinical summary accurate? (Not accurate: The clinical summary contains any content it should not contain) | [Yes/No/Partially] |
| Is the plan and requested actions section accurate? (Not accurate: The clinical summary contains any content it should not contain) | [Yes/No/Partially] |
| Which information is inaccurate? | [free-text] |
| Does the inaccuracy relate to a problem with: | [The source data/The system message or hallucination/Conflict with clinical guidelines or routine practice/Other] |
| If selected other in the previous question, please complete: | [free-text] |
| Provide details of the problem and suggest a solution E.g. Incorrect procedure due to error in ward round note, which states 'below knee amputation'. Operation note states 'above knee amputation'. Suggest amending prompt such that the output for surgical procedures is derived from the operation note. | [free-text] |
| What is the likely clinical significance of the missing or inaccurate data? | [Great clinical significance; little clinical significance; no clinical significance] |
| Readability of the discharge summary | [Clear & concise/ Clear but too long/Concise but unclear/Unclear and too long] |
| Just by considering the input data, would you, in real life, need to consult with the team that treated the patient? | [Yes/No] |
| **Global Bias Rating** | |
| Does the discharge summary output contain any information that is inaccurate or inapplicable for a particular demographic? E.g. patient gender, age, clinical specialty Provide details. | [Yes/No] |
| If selected "Yes" in the previous question, please provide details: |  |
| **Global Confidence Rating** | |
| Would you sign your name to this discharge summary? | [Yes/No/Yes with minor changes] |

^1^ © 2026 Imperial College Healthcare NHS Trust. All rights reserved. No part of this publication may be reproduced, distributed, or transmitted in any form or by any means, including photocopying, recording, or other electronic or mechanical methods, without the prior written permission of the Imperial College Healthcare NHS Trust (ICHT). For permission requests, write to ICHT at [imperial.DCS@nhs.net](mailto:imperial.DCS@nhs.net)

**Supplementary Table 2** Sample ICD-10 Diagnosis

| **ICD-10 Diagnosis Description** | **Count; n (%)** |
| --- | --- |
| Abnormal levels of other serum enzymes | <5 (<9) |
| Acute renal failure, unspecified | <5 (<9) |
| Angina pectoris, unspecified | <5 (<9) |
| Disorders of calcium metabolism | <5 (<9) |
| Endometrial glandular hyperplasia | <5 (<9) |
| Extended spectrum betalactamase (ESBL) resistance | <5 (<9) |
| Fracture of shaft of tibia | <5 (<9) |
| Gastroenteritis and colitis of unspecified origin | <5 (<9) |
| Hyperkalaemia | <5 (<9) |
| Hyperparathyroidism, unspecified | <5 (<9) |
| Intra-abdominal and pelvic swelling, mass and lump | <5 (<9) |
| Malignant neoplasm of ovary | <5 (<9) |
| Melaena | <5 (<9) |
| Neoplasm of uncertain or unknown behaviour: Brain, unspecified | <5 (<9) |
| Other and unspecified abdominal pain | <5 (<9) |
| Other congenital deformities of hip | <5 (<9) |
| Other disorders of nervous system, not elsewhere classified | <5 (<9) |
| Pneumonia, unspecified | <5 (<9) |
| Respiratory failure, unspecified | <5 (<9) |
| Sepsis, unspecified | 11 (19.0) |
| Spinal stenosis | <5 (<9) |
| Tendency to fall, not elsewhere classified | <5 (<9) |
| Unspecified injury of hip and thigh | <5 (<9) |
| Unspecified jaundice | <5 (<9) |
| Vitamin D deficiency, unspecified | <5 (<9) |
| Missing | 22 (37.9) |

* Sum of counts is 58 (larger than the sample size=52) as a patient could have more than one diagnosis within the same encounter.

**Supplementary Table 3** Table of Encounter Hospital Specialty

| **Hospital Speciality** | **Count; n (%)** |
| --- | --- |
| A&E | <5 (<7) |
| Anaesthetics | <5 (<7) |
| Clinical Pharmacology | <5 (<7) |
| Endocrinology | <5 (<7) |
| Gastroenterology | 10 (13.3) |
| General Medicine | 8 (10.7) |
| Geriatric Medicine | 8 (10.7) |
| Gynaecology | <5 (<7) |
| Neurosurgery | <5 (<7) |
| Obstetrics | <5 (<7) |
| Paediatrics | <5 (<7) |
| Respiratory/Thoracic Medicine | 5 (6.7) |
| Rheumatology | <5 (<7) |
| Trauma & Orthopaedics | <5 (<7) |
| Missing | 22 (29.3) |

* Sum of counts is 75 (larger than the sample size=52) as a patient could attend more than one speciality within the same encounter.

**Supplementary Table 4** Proportions and confidence intervals for secondary variables

Supplementary Table 4 Proportion of observations for the secondary outcomes across iterations 1 and 2 (development dataset) and the final evaluation (test dataset). Values are presented as n, percentage and 95% confidence intervals (Wilson method) presented as percentages. n indicates the total number of observations in each iteration excluding missing values. N/A indicates where the response option was not included in the evaluation questionnaire for that iteration.

| Secondary Variable | Iteration 1  n(%; 95% CI) | Iteration 2  n(%; 95% CI) | Test  n(%; 95% CI) |
| --- | --- | --- | --- |
| Is the clinical summary accurate? | 1 (n=43) | 2 (n=39) | test (n=9) |
| No | 4 (10;4-22) | 2 (5;1-18) | 2 (22;6-55) |
| Partially | 11 (26;15-41) | N/A | N/A |
| Yes | 27 (64;49-77) | 35 (95;82-99) | 7 (78;45-94) |
| Is the clinical summary complete? | 1 (n=43) | 2 (n=39) | test (n=9) |
| No | 4 (9;4-22) | 2 (5;1-17) | 1 (11;2-43) |
| Partially | 24 (56;41-70) | 0 (0;0-9) | 0 (0;0-30) |
| Yes | 15 (35;22-50) | 37 (95;83-99) | 8 (89;57-98) |
| Is the plan and requested actions accurate? | 1 (n=37) | 2 (n=39) | test (n=9) |
| No | 3 (8;3-21) | 10 (26;15-41) | 2 (22;6-55) |
| Partially | 17 (46;31-62) | 0 (0;0-9) | 0 (0;0-30) |
| Yes | 17 (46;31-62) | 29 (74;59-85) | 7 (78;45-94) |
| Is the plan and requested actions complete? | 1 (n=42) | 2 (n=39) | test (n=9) |
| No | 9 (21;12-36) | 8 (21;11-36) | 2 (22;6-55) |
| Partially | 17 (40;27-56) | 0 (0;0-9) | 0 (0;0-30) |
| Yes | 16 (38;25-53) | 31 (79;64-89) | 7 (78;45-94) |
| Degree of readability | 1 (n=43) | 2 (n=38) | test (n=9) |
| Clear & concise | 19 (44;30-59) | 31 (82;67-91) | 9 (100;70-100) |
| Clear but too long | 6 (14;7-27) | 3 (8;3-21) | 0 (0;0-30) |
| Concise but unclear | 15 (35;22-50) | 4 (11;4-24) | 0 (0;0-30) |
| Unclear and too long | 3 (7;2-19) | 0 (0;0-9) | 0 (0;0-30) |
| Is there any information that is inaccurate or inapplicable for a particular demographic? | 1 (n=43) | 2 (n=39) | test (n=9) |
| No | 40 (93;81-98) | 36 (92;80-97) | 7 (78;45-94) |
| Yes | 3 (7;2-19) | 3 (8;3-20) | 2 (22;6-55) |
| Are there any formatting issues or spelling errors? | 1 (n=25) | 2 (n=35) | test (n=8) |
| No | 25 (100;87-100) | 30 (86;71-94) | 7 (88;53-98) |
| Yes | 0 (0;0-13) | 5 (14;6-29) | 1 (12;2-47) |
| What is the clinical significance of missing or inaccurate data? | 1 (n=43) | 2 (n=39) | Test (n=9) |
| High risk of harm | 3 (7;2–19) | 1 (3;0–13) | 0 (0;0–30) |
| Moderate risk of harm | 12 (28;17–43) | 4 (10;4–24) | 1 (11;2–43) |
| Minor risk of harm | 13 (30;19–45) | 10 (26;15–41) | 5 (56;27–81) |
| No risk | 7 (16;8–30) | 6 (15;7–30) | 2 (22;6–55) |
| No missing or inaccurate data | 8 (19;10–33) | 18 (46;32–61) | 1 (11;2–43) |
| Just by considering the input data, would you, in real life, need to consult with the team that treated the patient? | 1 (n=0) | 2 (n=37) | test (n=9) |
| No | N/A | 28 (76;60-87) | 6 (67;35-88) |
| Yes | N/A | 9 (24;13-40) | 3 (33;12-65) |

**Supplementary Figure 1** Primary Outcome Stratified by (A) Length of Stay, (B) Surgical Admission and (C) A&E Admission. Bar charts are created after pooling results from the development dataset using GPT-generated discharge summaries from iteration 2 and the test dataset (final evaluation), where the three options “yes”, “yes with minor changes” and “no” were possible.
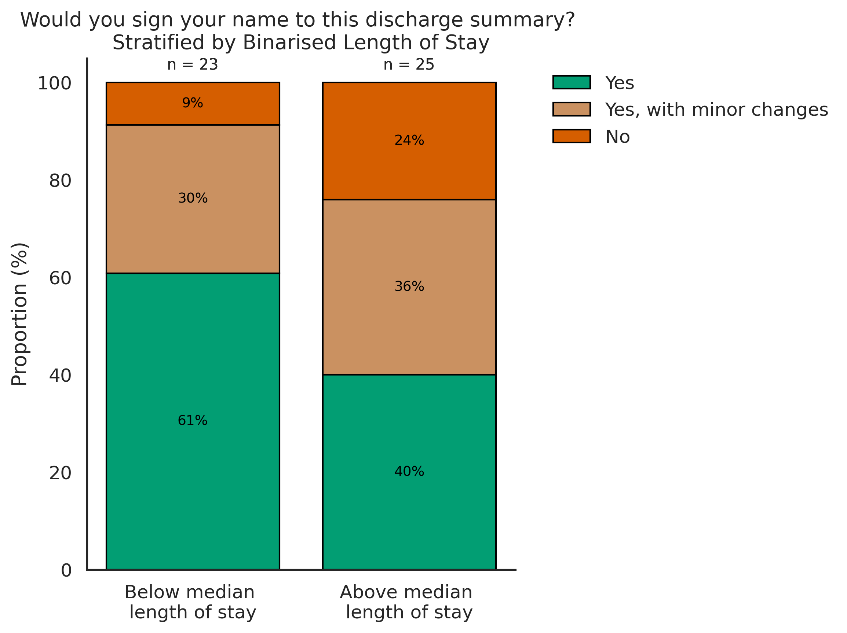

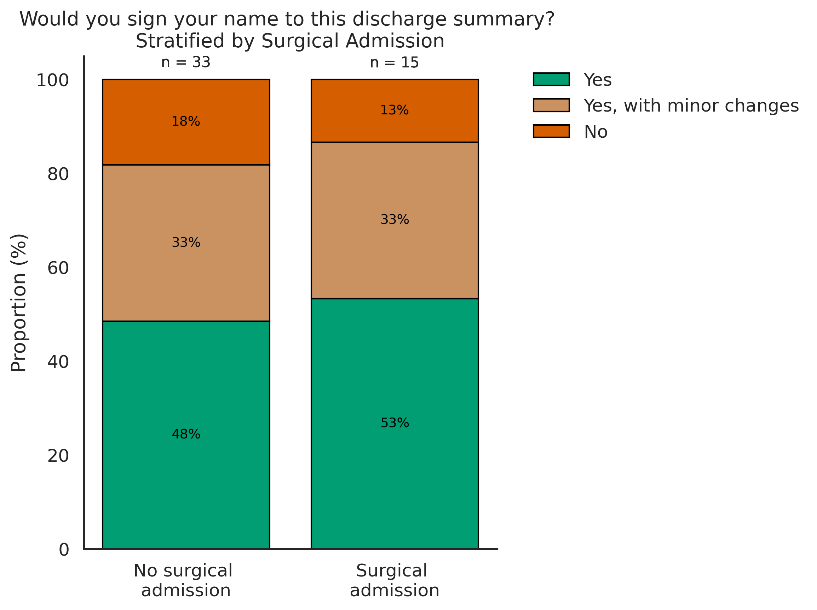

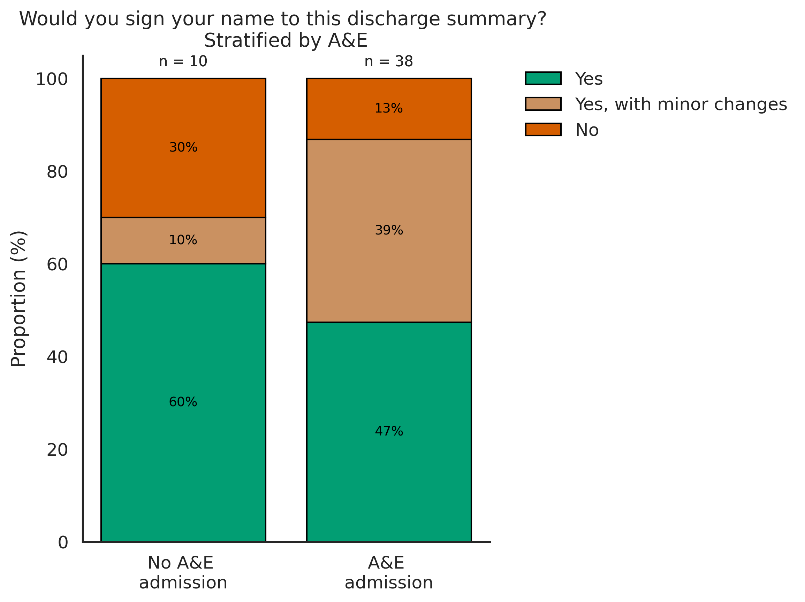


1. [↑](#endnote-ref-2)
